# Linking inter-individual differences in the perceptual load effect to spontaneous brain activity

**DOI:** 10.3389/fnhum.2015.00409

**Published:** 2015-07-23

**Authors:** Lu Liu, Jinfeng Tan, Antao Chen

**Affiliations:** Key Laboratory of Cognition and Personality of Ministry of Education, Faculty of Psychology, Southwest UniversityChongqing, China

**Keywords:** perceptual load effect (PLE), inferior temporal gyrus (ITG), precentral/postcentral gyrus, resting-state fMRI, amplitude of low-frequency fluctuation (ALFF)

## Abstract

Previous researches have widely demonstrated that the interference from peripheral distractor will decrease when the task load is high. However, no study to date has paid attention to the individual differences in perceptual load effect (PLE) and little is known of spontaneous brain activity associated with PLE during resting state. To investigate this issue, we used resting-state functional Magnetic Resonance Imaging (fMRI) to examine the relationship between the amplitude of low-frequency fluctuations (ALFFs) and PLE. The results showed that there were large individual differences in PLE and we found PLE was significantly associated with ALFFs in left inferior temporal gyrus (ITG) and left precentral/postcentral gyrus. The present study suggested that the PLE was measurable, and there were individual differences in this effect. Moreover, these results implicated that: 1) mutual competition for limited capacity, which is involved in visual attention, and 2) response control that is included in behavior response both may contribute to the modulation induced by perceptual load.

## Introduction

It is very important to ignore irrelevant stimuli in life. “Early selection” and “late selection” models of attention, which have different views on role of attention in information processing, has led to a longstanding debate on attention theory (Driver, 2001). Perceptual load theory not only offers a proper resolution to this early and late selection debate, but also clearly delineates the main determinants of successful focused attention and distractor ignorance: the level of perceptual load in current task (Lavie and Tsal, 1994; Lavie, 1995). According to perceptual load theory, perception has limited capacity and process automatically, in the low perceptual load tasks, any spare capacity not taken up in task-relevant stimuli will spill over involuntarily to task-irrelevant distractors, which lead to late selection. In contrast, tasks involving high perceptual load will run out full capacity on relevant stimuli, as a result irrelevant distractors have no chance to be perceived, which lead to early selection. In fact, load theory combines the early and late selections into a hybrid model (Lavie and Tsal, 1994; Lavie, 1995, 2005, 2010).

A series of behavior experiments demonstrated the critical role of perceptual load in determining the efficiency of task performance in face of distractions (Lavie, 2005, 2010). In a typical experiment situation, participants are asked to perform a central search task while ignore an irrelevant peripheral distractor that can be congruent, incongruent or neutral with the target. The perceptual load level was manipulated by changing the search numbers of nontarget letters around the target (Lavie, 1995; Lavie and Cox, 1997). The congruency effect (the difference of reaction time between incongruent and congruent conditions or the difference between incongruent and neutral conditions) is taken as interference effect from peripheral task-irrelevant stimulus. The perceptual load theory is typically demonstrated by the finding that the congruency effect is reduced when perceptual load is high relative to when it is low (Lavie, 2005). Functional magnetic resonance imaging (fMRI) studies further confirmed the effect of perceptual load on peripheral distractions (Rees et al., 1997; Schwartz et al., 2004; Yi et al., 2004; Bahrami et al., 2007). In these fMRI researches, participants were often asked to perform a relevant task in the central display under low load (LL) or high load (HL) conditions, and ignore the peripheral task-irrelevant distractors (e.g., moving dots, scene) which can activate certain areas (e.g., V5 area, parahippocampal place area). Response to peripheral task-irrelevant distractors in these areas was reduced when central relevant task load was increased, which is in accordance with the predictions of perceptual load theory.

Although convergent evidences indicate a clear modulation of perceptual load on interference effect, it is poorly understood about the individual difference of this modulation. Obviously, this issue is crucial for us to understand how perceptual load works. In the present study, we planned to investigate the individual difference of perceptual load effect (PLE), which is embodied in the decrease of interference effect from task-irrelevant distractors, with behavioral and fMRI measurements. Using a variation of visual search task (Lavie and Cox, 1997), we first examined the individual difference of PLE on the behavioral level. Further, we utilized the resting-state fMRI (RS-fMRI) to study the neural correlates of this individual difference. The RS-fMRI is a relatively new method that can be used to explore the intrinsic functional architecture of the brain based on measurements of spontaneous low-frequency (<0.08 Hz) fluctuations (LFFs) in the blood oxygenation level dependent signal (Biswal et al., 1995; Fox and Raichle, 2007; Raichle, 2010; Biswal, 2012). Moreover, physiological variances, such as neurovascular effect, would induce variability into blood oxygen level dependent (BOLD) activation results and decrease measurement accuracy of BOLD signal (Kannurpatti et al., 2011; Di et al., 2013; Kalcher et al., 2013). Endogenous and task-free resting-state amplitude of low frequency fluctuation (ALFF) could imply regional spontaneous neuronal activity while reduce the neurovascular effect, which is a complementary method for task-evoked fMRI. The correlation analyses between RS-fMRI data and behavioral performances could straightforwardly reflect the spontaneous brain activity bases of these performances (e.g., Bing et al., 2013; Pan et al., 2014). Therefore, the RS-fMRI has a unique advantage to investigate the underlying neural basis of PLE while reduce variability of physiology factors.

In the RS-fMRI studies, the amplitude of spontaneous low-frequency fluctuations (ALFFs) has been widely used for measuring the intensity of regional spontaneous fluctuations in brain activity (Zang et al., 2007; Zou et al., 2008; Wang et al., 2013). Previous studies have demonstrated that ALFF analyses have robust predictive value for behavior and can be used to investigate the neural basis of individual differences in behavioral performance (Yang et al., 2011; Wei et al., 2012; Takeuchi et al., 2013; Pan et al., 2014; Wang et al., 2014). In present study, we attempted to explore the intrinsic neural basis of PLE by calculating the ALFF value of RS-fMRI signals and correlating it with PLE in behavior performance.

The task-based neuroimaging studies about visual search tasks provide important cues for the spontaneous brain activity bases of PLE. Especially, in previous studies examining the neural correlates of visual attention (Desimone and Duncan, 1995; Donner et al., 2002; Nobre et al., 2003), the activations of some fronto-parietal regions, including superior and inferior parietal lobules, intraparietal sulcus, dorsolateral prefrontal and premotor areas, were often observed. Moreover, the inferior temporal (IT) cortex was activated when the attention was being biased (Chelazzi et al., 1993, 1998; Desimone, 1996), reflecting a top-down modulation of the attention system on visual processing. Therefore, we assumed that the individual differences in PLE may relate to ALFF values in areas mentioned above.

## Materials and Methods

### Participants

Forty six healthy college students (17 males, 29 females, aged 17–20 years), with no history of neurological and psychiatric disorders, were recruited for the study with a compensation. All participations had normal or corrected-to-normal vision. This study was approved by the Southwest University Human Ethics Committee for the Brain Mapping Research, and written consent was obtained from each subject before scanning.

### Stimuli and Procedure

**Figure 1:**
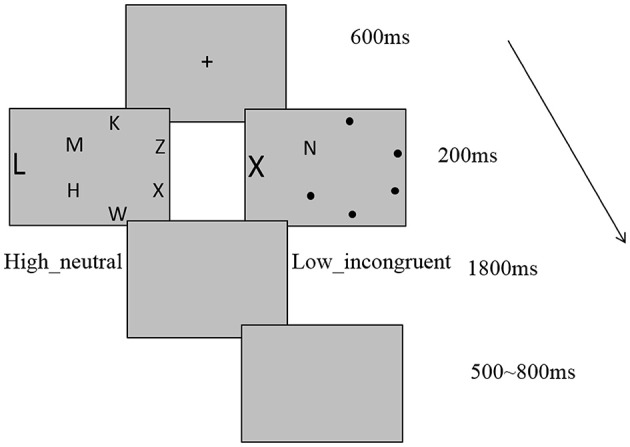
**Example of trial sequence and example display with high load neutral and low load incongruent conditions**.

Figure 1 depicts the trial sequence and example display. Participants performed a perceptual load task in our experiment. The task display in each trial consisted of a letter circle centered at fixation, plus a peripheral distractor letter, presented to the left or right of circle. The search targets were X and N. Each circle contained one target, and subjects were instructed to indicate which of the target letters was present in the circle by pressing either the “1”or the “2” key on the computer as quickly as possible while not sacrificing accuracy. The peripheral distractor letter was equally likely to be T or L and subjects were instructed to ignore the distractor. In the HL condition, the letters H, M, K, Z, and W were placed randomly in the nontarget circle positions in a different order on each trial. In the LL condition, there were small black points in nontarget positions. The peripheral distractor letter was either “T” or “L” in neutral condition and was the same as the other search target in incongruent condition. Target position, distractor position and identity, and their combinations were counterbalanced across subjects. Stimuli were presented on a gray background. Each trial started with the presentation of a central cross for 600 ms. After the task display had been displayed for 200 ms, there was a gray blank screen for 1800 ms, which was the response period, followed by an additional 500–800 ms gray blank screen that acted as the inter-trial interval (ITI). Our experiment comprised of four blocks. The first block was practice block, which contained 24 trials. The other three blocks were formal experiment blocks, each consisted of 96 trials presented in a pseudo-random order within each block.

### MRI Data Acquisition

Images were acquired with a 16-channel head coil Siemens 3T scanner (Siemens Magnetom Trio TIM, Erlangen, Germany), an echo-planar imaging (EPI) sequence was used for data collection, and 240 T2*-weighted images were recorded per run (TR = 2000 ms; TE = 30 ms; 33 slices, slice thickness = 3 mm; gap = 0.6 mm; flip angle = 90° field of view (FOV) = 200 × 200 mm^2^; image matrix, 64*64). In addition, one single T1-weighted volume was measured (TR = 2530 ms; TE = 3.39 ms; 128 slices, slice thickness = 1.33 mm; flip angle = 7° inversion time = 1100 ms; FOV = 256 × 256 mm^2^; image matrix, 256*256). Each subject first underwent a brief resting-state scan during which they were instructed to relax with their eyes closed and not to think of anything in particular, then subjects completed perceptual load task.

### MRI Data Preprocessing

The imaging data preprocessing was carried out by using Data Processing Assistant for Resting-State fMRI (DPARSF; Chao-Gan and Yu-Feng, 2010), which is based on Statistical Parametric Mapping (SPM8)^1^ and Resting-State fMRI Data Analysis Toolkit (Song et al., 2011). In order to get magnet-steady images, the first 10 volumes were discarded. Then slice timing and realign were performed, participants exceeding 2.5 mm of translation and 2.5° of rotation were discarded. The fMRI data of one subject was excluded due to excessive head movement. Next the functional scans were normalized to the standard Montreal Neurological Instituted space, resampled to 3 × 3 × 3 mm^3^. Then we conducted the spatial smoothing with 4-mm full width at half maximum (FWHM) Gaussian kernel. Band-pass filtered (0.01–0.8 Hz) was applied after ALFF calculation to reduce the influences of low-frequency drift and high frequency noise.

### ALFF Calculation and ALFF-Behavior Analysis

The ALFF analysis was performed by using Resting-State fMRI Data Analysis Toolkit (Song et al., 2011). First we got time series after preprocessing, and then time series of every voxel was transformed to a frequency domain with a fast Fourier transform (FFT) to obtain the power spectrum. Then the square root was calculated at each frequency of the power spectrum and was averaged across 0.01–0.08 Hz at each voxel. This averaged square root was taken as the ALFF. For the standardization purpose, we calculated the mean ALFF within the gray matter mask for each participant, and the ALFF value of each voxel was divided by this mean value.

In order to examine whether ALFF was related to the PLE, we made a correlation analysis between ALFF and residualized PLE (we calculated Pearson correlation coefficient between ALFF and PLE which had regressed out flanker interference scores, age, gender and standard deviations of RT (SD_RT_) within a brain mask (70831 voxels). PLE index:

$$\text{PLE=}\frac{{\text{IE}}_{\text{LL}}{\text{−IE}}_{\text{HL}}}{\text{Mean RT}}$$

In the formula, IE is interference effect from distractor, LL is low load condition, HL is high load condition. PLE actually reflected reduction of flanker interference effect on the HL condition compared with LL condition, and this reduction was divided by the mean reaction time for standardization purpose.

## Results

### Behavioral Data

**Figure 2:**
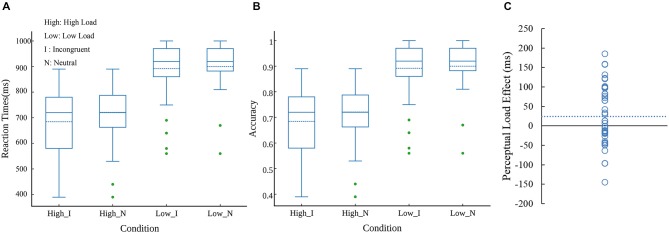
**Distributions of reaction times **(A)** and accuracy **(B)** at four conditions**. The dash line in boxes reflect mean value. Note: High, high load; Low, low load; *I*, incongruent; *N*, neutral. Individual differences related to PLE (without normalization) **(C)**. Each circle represents one participant’s score in PLE. The dash line reflect mean value.

Mean RTs, accuracy rates, kurtosis, skewness and W value were presented in Table 1. As shown in Table 1, kurtosis and skewness of the task were acceptable for the normality assumption, with the range between −1 and 1 (Marcoulides and Hershberger, 1997). The correct response times on the perceptual load task were analyzed in an ANOVA with task load (low perceptual load and high perceptual load) and congruency (incongruent and neutral) as within-subjects factors. The result showed that main effect of task load was significant (*F*_(1,41)_ = 163.907, *p* < 0.0001, ${\text{η}}_{\text{p}}^{2}$ = 0.79); the interaction between these two factors was also significant (*F*_(1,41)_ = 4.88, *p* = 0.0328, ${\text{η}}_{\text{p}}^{2}$ = 0.1); however, the main effect of congruency was not significant. In the LL condition, participants responded slowly in the incongruent trials compared with neutral trials (*t*_(44)_ = 2.442, *p* = 0.0187, 95% confidence interval [1.72, 17.95]). In the HL condition, there was no significant difference between the incongruent and neutral trials. Specifically, the interference effect from distractor in HL condition was significantly less than that in LL condition (*t*_(44)_ = −2.209, *p* = 0.0324, 95% confidence interval [−47.46, −2.18]). These results suggested a significant modulation of perceptual load on congruency effect. Figure 2A displayed distribution of reaction times at each condition. However, as shown in Figure 2C, there was a substantial amount of inter-individual variability in PLE (without normalization).

**Table 1:** **The statistics for response times and accuracy rates from behavioral performance data by task load and distractor congruency**.

	Congruency		
Task load	*I*	*N*	*I*–*N*
Low
RT, SD_RT_ (ms)	605.23 (67.35)	595.40 (80.65)	9.83 (27.01)
Accuracy, SD_Accuracy_	89.26% (11.61%)	90.06% (9.20%)	
Kurtosis	0.75	0.89	0.35
Skewness	−0.71	−0.91	
High
RT, SD_RT_ (ms)	838.80 (182.01)	853.79 (187.33)	−14.99 (70.02)
Accuracy, SD_Accuracy_	68.46% (12.41%)	72.10% (11.22%)	
Kurtosis	0.34	1.00	−0.57
Skewness	−0.67	−0.69	
PLE			
RT, SD_RT_ (ms)			24.82 (75.37)
Kurtosis			−0.55

*I, incongruent; *N*, neutral; Low, low load; High, high load*.

Participants were more accurate in LL condition than in HL condition (*F*_(1,41)_ = 264.14, *p* < 0.0001, ${\text{η}}_{\text{p}}^{2}$ = 0.86), and were more accurate in neutral condition than in incongruent condition (*F*_(1,41)_ = 6.49, *p* = 0.0147, ${\text{η}}_{\text{p}}^{2}$ = 0.13), the interaction between perceptual load and congruency was not significant. However, according to Figure 2B that displayed distribution of accurate rate at each condition, we removed three outliers, the interaction effect was significant (*F*_(1,41)_ = 4.42, *p* = 0.0417, ${\text{η}}_{\text{p}}^{2}$ = 0.1) as well.

### ALFF-Behavior Correlation Analysis

**Figure 3:**
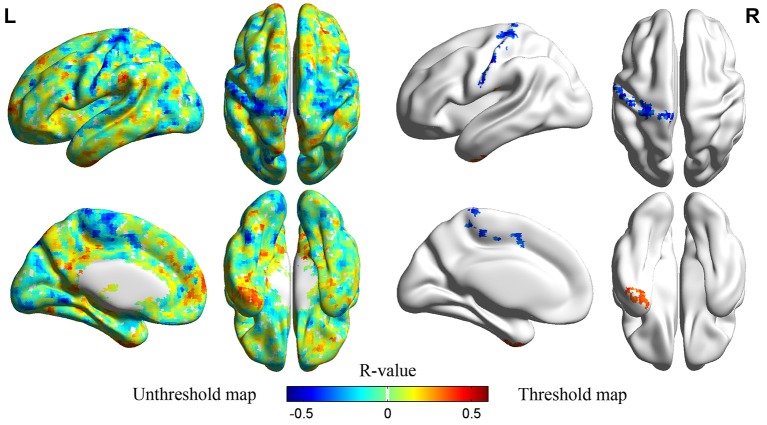
**Left unthreshold map showed correlations between averaged ALFF values and PLE without multiple comparison correction**. The right threshold map displayed brain regions that exhibited significant correlations between averaged ALFF values and PLE (left to left, right to right). The threshold was set at *p* < 0.05 (corrected by AlphaSim).

**Figure 4:**
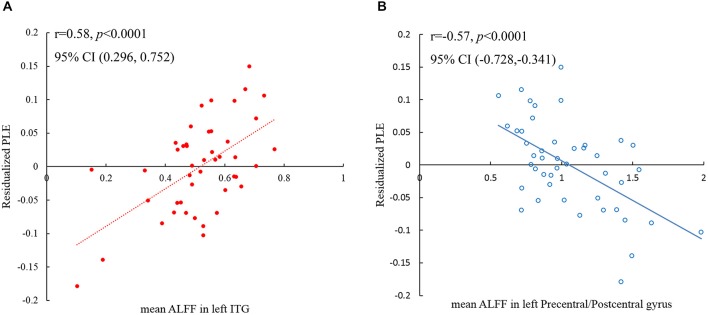
**Scatter plot shows the Pearson correlation between PLE and averaged ALFF in left ITG (A) and left precentral/postcentral gyrus (B) respectively, while the age, gender, SD_RT_ and distractor interference scores were regressed out from the PLE scores**. Each dot in **(A)** and each circle in **(B)** represents data from one participant.

PLE significantly correlated with ALFF values in left IT gyrus (ITG) and left precentral/postcentral gyrus at a threshold of *p* < 0.05 (single voxel, *p* < 0.05, cluster size ≧2295 mm^3^), corrected by AlphaSim^2^ (Figure 3; Table 2). Scatter plot displayed the relationship between residualized PLE and mean ALFF in ITG (Figure 4A) and precentral/postcentral gyrus (Figure 4B), which were measured with Pearson correlation coefficient. Moreover, we also investigated ALFF-behavior association using Spearman correlation that was a more robust measure reported in previous study (Rousselet and Pernet, 2012). The Spearman correlation coefficient between residualized PLE and mean ALFF in ITG was 0.456, *p* = 0.0015, 95% confidence interval [0.183 0.659], while was −0.519 in precentral/postcentral gyrus, *p* = 0.0003, 95% confidence interval [−0.726 −0.234]. Confidence intervals for Pearson and Spearman correlation values were estimated using a percentile bootstrap.

**Table 2:** **Brain regions showed significant ALFF-PLE correlations across subjects**.

Region	BA	No. voxels	Peak *r*	*x*	*y*	*z*
L. Inferior Temporal Gyrus	20	102	0.58	−36	−15	−39
L. Precentral/Postcentral Gyrus	3/6	198	−0.57	−9	−36	72

## Discussion

Combining a modified visual search task and an expression calculating the modulation effect of perceptual load, we first evaluated the individual differences in PLE, and then investigated the neural correlates of PLE through ALFF-behavior correlation analysis. The behavioral results showed that there was a substantial amount of individual variability in PLE. Importantly, the ALFF-behavior correlation analysis indicated that the ALFF values significantly correlated with the PLE in brain areas left ITG and left precentral/postcentral gyrus. To our knowledge, this is the first time to quantify the modulation effect of perceptual load on visual selective attention, and explore the intrinsic brain functional architecture associated with the PLE.

Usually, the PLE was evaluated by the interaction between perceptual load and congruency effect. Nevertheless, the value of PLE was never calculated. In the present study, we aimed to investigate the individual difference of PLE, where it is necessary to calculate PLE for each participant. According to the perceptual load theory (Lavie and Tsal, 1994; Lavie, 1995), the PLE could be operationally defined as the reduced interference effects from distractors on high perceptual load condition compared to low perceptual load condition. On this basis, we put forward a simple expression to calculate PLE. The values of PLE are nearly normal distribution across participants, suggesting that this expression can validly measure the modulation effect of perceptual load. Moreover, the ALFF values in left in ITG and precentral/postcentral gyrus, which are implicated in visual selective attention, significantly correlated with PLE, further demonstrating the validity of this expression. Of course, the arithmetic for PLE needs to be verified and optimized in the future.

IT area is considered to be the final stage of the ventral visual processing pathway (Kravitz et al., 2013), and is critical to interpret the meaning of visual stimuli and establish object recognition (Tanaka et al., 1991; Logothetis and Sheinberg, 1996; Haxby et al., 2000). Visual processing of multiple items is constrained by capacity limitation, which may lead to mutual inhibitory interaction among stimuli for competing neural representation. Interestingly, Miller et al. (1993) found that presentation of multiple stimuli, relative to single stimuli, resulted in a suppression of the neuron responses in IT cortex, suggesting that activation in IT cortex reflects the competition for limited capacity. Accordingly, we propose that the activity of ITG at rest may index the intrinsic processing capacity for object perception and recognition, which may be reflected on the spontaneous activity observed in the present study. In accordance with this speculation, our results showed that the individual difference of ALFF values in ITG significantly associated with PLE. Furthermore, the result indicated that capacity limitation and more competition among stimuli in ITG may be the reason why the interference from peripheral distractor was decreased in high perceptual load condition.

Activations in precentral and postcentral gyrus were often found to be associated with response control (Liddle et al., 2001; Menon et al., 2001; Schumacher et al., 2003), suggesting that they contribute to controlling on response conflict during response output. When the ALFF in the two areas is high, their activities are also high at rest. Then, those participants with high ALFFs in precentral and postcentral gyrus should have strong ability to control response conflict. Further, the interference was small for the individuals whose ALFFs in precentral and postcentral gyrus are high. Because the interference was significantly present at low perceptual load, but eliminated at high perceptual load, from the expression of PLE, the value of PLE was virtually determined by the interference at low perceptual load. Thus, in the individuals with high ALFFs in percentral and postcentral areas, the PLE values are low because of the strong control on response conflict. As a result, the correlation between the ALFFs in precentral and postcentral gyrus and PLE is negative. Moreover, this result indicated that combined with less resources allocated to peripheral distractor, stronger control for response conflict contributed to decrease of distractor interference in high perceptual load.

In conclusion, our study quantified PLE and supplied evidence of individual difference in brain spontaneous activity linked to PLE. We found two regions that were significantly associated with PLE, including ITG and precentral/postcentral gyrus. The present findings indicated that capacity limitation involved in visual attention and response control involved in top-down attention control had close relation with PLE. Furthermore, task-based fMRI studies could increase its measurement accuracy with ALFF measurement (Kalcher et al., 2013), which give implications for further studies of PLE. Combine task-based fMRI with resting-state fMRI, such as ALFF, may make us have a deeper insight into this issue.

## Conflict of Interest Statement

The authors declare that the research was conducted in the absence of any commercial or financial relationships that could be construed as a potential conflict of interest.

## Footnotes

^1^http://www.fil.ion.ucl.ac.uk/spm

^2^See the AlphaSim command description at http://afni.nimh.nih.gov/afni/doc/manual/AlphaSim
